# Effect of BMP-2 Adherent to Resorbable Sutures on Cartilage Repair: A Rat Model of Xyphoid Process

**DOI:** 10.3390/ma13173764

**Published:** 2020-08-26

**Authors:** Nathan Drummond, Bradley W. Bruner, Michael H. Heggeness, Bradley Dart, Shang-You Yang

**Affiliations:** 1 Department of Orthopaedic Surgery, University of Kansas School of Medicine-Wichita, Wichita, KS 67214, USA; nathan.drummond@utexas.edu (N.D.); bbruner@ksjointspine.com (B.W.B.); heggeness@msn.com (M.H.H.); bdart@kumc.edu (B.D.); 2 Department of Biological Sciences, Wichita State University, Wichita, KS 67260, USA

**Keywords:** resorbable suture, BMP-2, meniscal repair, xyphoid process, animal model

## Abstract

Meniscal tears are often seen in orthopedic practice. The current strategy for meniscal repair has only had limited success with a relatively high incidence of re-operative rate. This study evaluates the therapeutic effects of Bone morphogenetic protein-2 (BMP-2) soaked sutures for cartilage repair, using a rat model of xyphoid healing. Vicryl-resorbable sutures were presoaked in BMP-2 solutions prior to animal experimentation. Rat xyphoid process (an avascular hyaline cartilage structure) was surgically ruptured followed by repair procedures with regular suture or with sutures that were pre-soaked in BMP-2 solutions. In vitro assessment indicated that presoaking the Vicryl-resorbable sutures with 10 µg/mL BMP-2 resulted in a sustained amount of the growth factor release up to 7 days. Histological analysis suggested that application of this BMP-2 soaked suture on the rat xyphoid process model significantly improved the avascular cartilage healing compared to non-soaked control sutures. In conclusion, data here confirm that the rat xyphoid process repair is a reproducible and inexpensive animal model for meniscus and other cartilage repair. More importantly, coating of BMP-2 on sutures appears a potential avenue to improve cartilage repair and regeneration. Further study is warranted to explore the molecular mechanisms of this strategy.

## 1. Introduction

Arthroscopy of the knee for meniscal pathology is a very common orthopedic procedure performed in the United States. Although the most common treatment for torn meniscal tissue is partial meniscectomy, over 300,000 arthroscopic meniscal repairs are conducted in the USA annually [1]. Since the early stages of knee arthroscopy, meniscectomy remains the dominant treatment for meniscal tears. However, a substantial amount of research has suggested that meniscectomy negatively affects the stability and longevity of the knee joint, resulting in deficiencies in knee performance [2,3,4,5,6]. With removal of a part of the meniscus, the total contact area between the femur and tibia is decreased and the smaller area of tibiofemoral contact bears much higher pressures [3]. This can alter the knee mechanics and lead to earlier onset of osteoarthritis. The mounting evidence of negative long-term results following meniscectomy has led to increasing research in meniscal repair. Unfortunately, meniscal repair has only had limited success [6,7], and the re-operative rate following meniscal repair remains high. The further from the periphery of the meniscus, the less likely a meniscal repair is to heal. Tears over 4mm from the periphery of the meniscus are unlikely to heal with repair [8].

It is generally believed that meniscal repairs in younger patients have a higher likelihood of healing, but this is controversial. Additionally it has been shown that repairing a torn meniscus at the same time as an anterior cruciate ligament (ACL) reconstruction results in significantly greater incidence of meniscal healing [9]. This is thought to be due to the release of blood and cytokines during ACL reconstruction. Indications for meniscal repair often include: tears greater than 1 cm and less than 4 cm in length; vertical tears; red–red zone tears; patients under 40; tears less than 6 weeks old; normal mechanical axis of the knee; concurrent ACL reconstruction [5]. In order to increase the likelihood of healing meniscal repairs, several complimentary procedures have been proposed and tested. Abrasion of the meniscus using either a rasp or arthroscopic shaver has been shown to increase the expression of cytokines [10] and in practice has led to higher healing rates [11]. Trephination technique is also available, which creates channels from vascular to avascular portions of the meniscus using a spinal needle. Finally, autologous platelet rich plasma, which has an increased level of multiple growth factors has been suggested to improve the rate of healing when injected near the meniscal repair [12].

Bone morphogenetic protein-2 (BMP-2) belongs to the transforming growth factor (TGF-β) superfamily that has been extensively investigated for its therapeutic roles in promoting bone and cartilage repair and regeneration by stimulating chondrogenesis [13,14,15,16]. Indeed, BMP-2 has been an Food and Drug Administration (FDA) approval biological agent for clinical use [17,18]. Since meniscal rupture is fibrocartilage damage, recent studies have been performed to examine BMP-2 stimulated progenitor cells for meniscus repair and regeneration [19,20,21,22]. We intended to investigate the local delivery of BMP-2 to the cartilage lesion site by soaking on the sutures. The delivery of growth factors to local tissues using coated sutures has been proposed as an innovative technique [23] and a preliminary study using vascular endothelial growth factor (VEGF) has been conducted in an ovine model [24]. Although the results did not indicate marked improvement, it was noted that growth factors could adhere to the suture material and that this was an effective means of growth factors to local tissues.

Meniscal repair, when successful, greatly improves the long-term functional outcome in patients with meniscal tears [1]. The barriers to widespread use of this procedure stem from the healing uncertainty of the repaired meniscal tissue. Adjunctive treatments to enhance healing of the repair will be of great benefit in making meniscal repair a more widely applicable procedure. For this to occur, a reliable animal model for human meniscus repair needs to be established. It has proven difficult to establish an accurate animal model of meniscal repair due to anatomical differences, in terms of microstructure, physiology, and material properties between humans and quadrupeds. There is currently no evidence that one animal model is best for the study of meniscal biology [2]. Therefore, considerable benefits may be derived from the development of a simple, inexpensive model to study cartilage repair and novel tissue engineering strategies for meniscal repair in rats.

This study proposed the development of an entirely novel model for the repair of cartilage tears using the laboratory rat; since rats usually respond to human growth factors, such a model might form an excellent screening platform. Previous models of meniscal tears have used large animals (sheep, pigs, goats, and dogs) [10], which incur major costs and require large animal facilities. The proposed model also has relevance to other cartilage repair procedures. The development of a rat model opens the potential to investigate the role of specific gene systems in cartilage repair, through the employment of transgenic rats (with deficiencies in or over expression of relevant chondrocyte regulatory factors).

## 2. Materials and Methods

**Materials.** The 4–0 absorbable sutures (VICRYL, Ethicon, Somerville, NJ, USA) and recombinant human BMP-2 (Infuse™, Medtronic, Minneapolis, MN, USA) were purchased through local medical supply (Ascension Via Christi Health). Biodegradable polymers poly-DL-lactide (PDLLA) were obtained from Sigma-Aldrich (St. Louis, MO, USA). All other chemicals and cell culture reagents are from ThermoFisher Scientific, in analytic or tissue culture grade.

**Fabrication of BMP-coated Sutures.** Sutures were coated using a modification of the technique of Fuchs et al. [23] for surgical implants. In total, 100 mg poly-DL-lactide (PDLLA) was dissolved in 1.5 mL of ethyl acetate at room temperature followed by filter sterilization. rhBMP-2 (Medtronic, Minneapolis, MN, USA) was mixed with various amounts of the PDLLA solution to obtain two concentrations of the growth factor (1 and 10 µg/mL). Sterile 4–0 Vicryl absorbable sutures were soaked into the coating solutions for 24 h at room temperature followed by air dry in a laminar flow hood.

**In vitro BMP-2 Release.** The BMP coated sutures at 1 cm in length were put in sterile Eppendorf tubes with 1 mL of phosphate-buffered saline (PBS) and incubated at 37 °C for 24 h. The incubation fluids were collected, and fresh PBS were replaced into the suture-containing tubes. The same procedures were repeated at day 4, 7, 14, and day 21. BMP concentrations in the incubation fluids collected were determined by ELISA, as described previously [25].

**Rat Xyphoid Model and Grouping.** The animal procedure was approved by the Institutional Animal Care and Use Committee (IACUC). A total of twelve female Lewis rats weighing approximately 200 g (age ~10 weeks) were used in this study. The animals were housed two in a cage, quarantined for at least one week before experimentation. They were randomly assigned to 4 groups (n = 3 for each group): two groups were treated with BMP coated suture and 2 groups were treated with standard suture. One group with each type of suture was sacrificed at 9 days and one at 21 days for histologic analysis of xyphoid healing.

**Figure 1:**
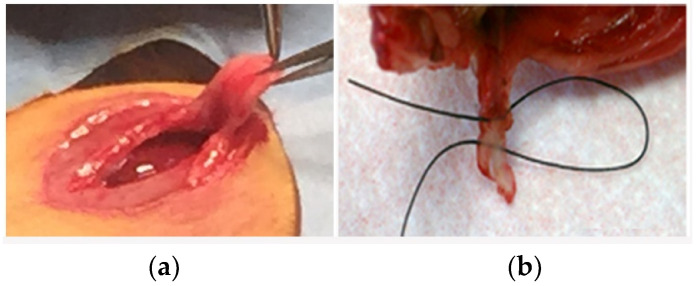
(**a**) Surgical tear of the xyphoid cartilage tissue on the model of cartilage repair; (**b**) suturing the xyphoid tissue with the BMP-coated suture.

**Surgical Procedure.** On the operation day, 0.05 mg/kg of Buprenorphine and 5 mg/kg of Carprofen were subcutaneously injected 1 h before surgery for preventative analgesia. Rats were anesthetized with intraperitoneal injection of a mixture of Xylazine (5 mg/kg) and Ketamine (50 mg/kg). Sterile ophthalmic ointment was applied to the eyes to prevent drying of corneas during surgery. An area of the ventral skin (2 cm^2^) was shaved using an electric animal clipper on the preparation table and sterilized by scrubbing with Povidone-iodine followed by application of 2% Chlorhexidine. A sterile paper drape was used to cover the body with the surgical site exposed through a small hole. A central skin incision was made along the midline over the base of the sternum, to expose the xyphoidxyphoid process. Fine scissors were then used to divide the xyphoid process along the midline and create a sufficiently large (~5 mm) surgical tear in an inferior to superior direction. This tear was then repaired with a single surgical loop passed and returned in a medial to lateral direction and secured with a surgical slip knot. The repaired suture was either BMP-2 coated or an uncoated suture (Figure 1) depending on the BMP-2 coated experimental group or uncoated control group. Previous experience indicated that the xyphoid process tear would not heal if no suture was given to hold the rupture together, therefore the non-suture control group was eliminated. The surrounding tissues were then closed with absorbable sutures. The wound was cleaned and rinsed with PBS containing 50 mg/mL Ampicillin to prevent local infection. To prevent premature suture removal by the animals, the rats were singly housed with Elizabethan collars for up to 3 days after surgery. Post-operative pain was properly controlled by 5 mg/kg Carprofen subcutaneous injections every 24 h, as indicated. At 9 and 21 days post-operatively, the rats were sacrificed and the xyphoid process was excised.

**Histological Process and Assessment.** Xyphoid process sections were cut along a horizontal axis, mounted and stained with hematoxylin & eosin (H&E) and toluidine blue. Sections were taken and stained along every 0.2 mm of the xyphoid process. Representative slides from the proximal, middle and distal thirds of each animal were chosen by a pathologist for analysis. In a blinded fashion, 5 investigators independently examined the histologic slides and made a determination of the healing present. Healing was graded from 0–5. A score of 0 represented no healing, 1 represented between 0 and 25% healed, 2 represented between 25 and 50% healed, 3 represented between 50 and 75% healed, 4 represented between 75 and 99% healed and 5 represented complete healing.

**Statistical Analysis.** The histological score results from all examiners were averaged. Results were analyzed by the Mann-Whitney statistics testing procedures using the SPSS software (version 22.0, IBM, Chicago, IL, USA). The level of significant difference was defined as *p* < 0.05.

## 3. Results

**Figure 2:**
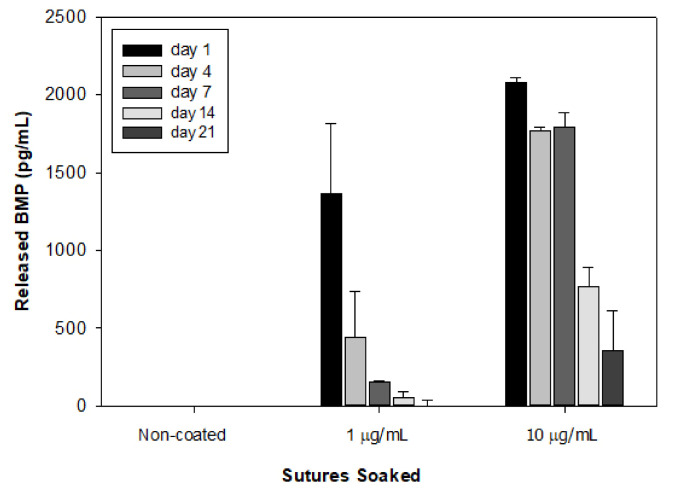
In vitro release assay of BMP-2 concentrations in elution at day 1, 7, 14, and 21 collections.

In vitro BMP-2 release from the coated sutures suggested that the 10 µg/mL concentration coated more BMP-2 onto the suture that sustained an amount of the growth factor and was released to the eluting solution up to 21 days (Figure 2). The sutures soaked in a low dose of BMP-2 quickly released all growth factor in the early days, while the sutures with 10 µg/mL BMP-2 coating presented a sustained release at reasonable clinical range up to 21 days. Therefore, sutures soaked in 10 µg/mL of BMP-2 were used for animal investigation.

**Figure 3:**
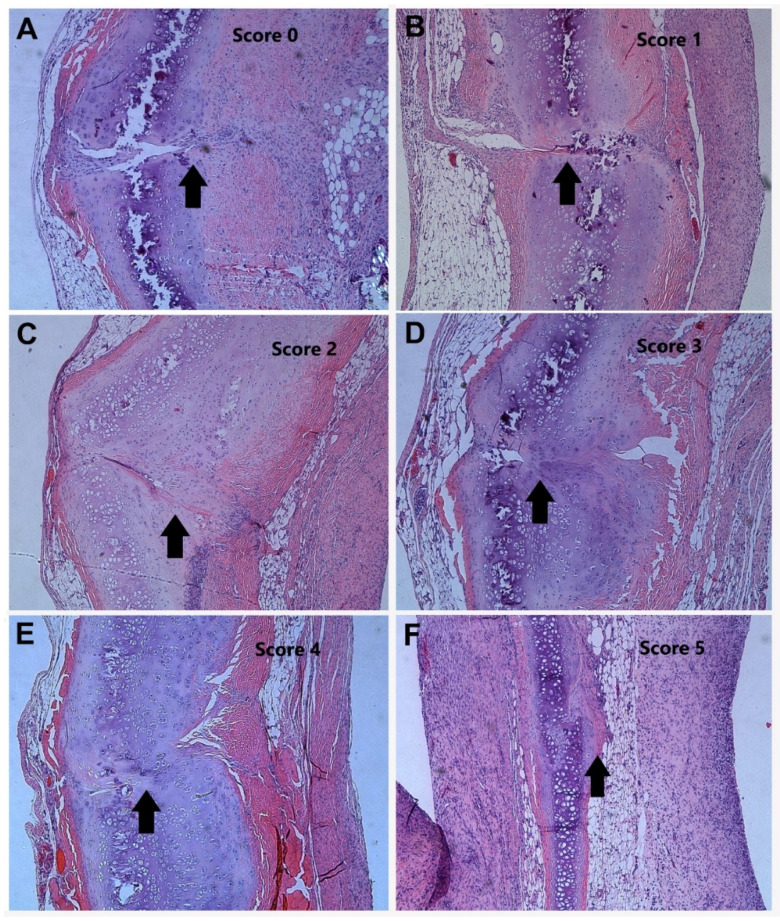
Typical examples of xyphoid cartilage tissue sections (H&E staining, 40×), representing the cartilage healing scores from 0–5 (**A**–**F**). Arrows indicate the cartilage healing.

**Figure 4:**
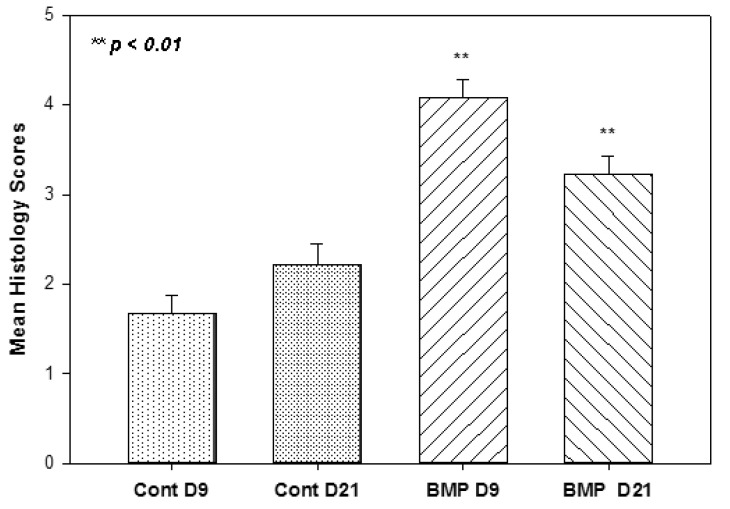
Average histology healing scores among non-coated (control) and BMP2-coated (BMP) groups at day 9 (D9) and day 21 (D21) after surgery.

All animals sustained surgery well and kept daily activity since recovery of anesthesia. No infection and other complications were noticed. Histology sections with H&E staining were blindly examined by the five investigators and the healing scores from the proximal third of the xyphoid were summarized. There was formation of fibrous tissue seen in all groups and new cartilage formation, to varying degrees, in most samples. Figure 3 illustrated representative examples of the histological appearance of the xyphoid cartilage tissues at different healing stages, from no cartilage healing (histological score 0) to complete healing (score 5). Quantification of the average healing among groups indicated that significantly better tissue healing was seen in the animals with BMP-coated suturing at both day 9 and day 21 groups, with dramatically higher histological scores than the counterparts in the control groups (Figure 4). There was no statistical difference between day 9 and 21 control groups. However, there was a statistically significant difference in the healing seen when comparing the control group day 9 to BMP day 9 (*p* < 0.001), and the control group day 21 to BMP day 21 (*p* < 0.004).

## 4. Discussion

The objective of the investigation tends to characterize an animal model to investigate the therapeutic influence of BMP-2 local release from soaked sutures for meniscal repair. The accomplishment of this study addresses several important goals. First, it shows the success of the rat xyphoid process cartilage model. Arnoczky et al. [2] believed that research effort at pre-clinical settings often provides the proof of principle of treatment options to advance the clinical practice. Appropriate animal models are critical in testing the efficacy and safety issues of the novel concepts of potential clinical treatment during the translational process. There is currently no appropriate animal model available in the literature for meniscal research; consequently, the rat xyphoid process model appears a good fit to test out our hypothesis in this experiment. It is recognized that the laboratory rat represents an effective means to develop novel pharmaceutical products and biological response modifiers. The rat responds to most human cytokines, chemokines and growth factors, and therefore provides a useful test platform for the development of novel strategies [26]. However, rats are typically not considered useful in the development of surgical procedures, as size limitations prohibit most surgical interventions. While the rat meniscus is beyond feasibility for manipulation, the rat xyphoid process is a relatively large body of avascular cartilage that does not ossify and is highly accessible through a surgical approach. With direct exposure of the xyphoid process through a single incision, this procedure was very easy to perform, and the animals returned to normal activities immediately after recovery from the anesthesia. Histologic analysis of the xyphoid does in fact reveal it to be an avascular cartilage structure that resembles some clinical pathologies of meniscal tear, thus it appears a suitable surrogate for studying avascular cartilage structures.

This study has also provided additional insight into the capability to coat sutures with growth factor material. Stimulating tissue healing with growth factors such as BMP-2 is a difficult process because of the short half-life of biological agents. For this reason, it seems more effective to administer them locally rather than systemically. Fuchs et al. [23] reported an investigation of insulin-like growth factor-1 (IGF-1) coated sutures for colon anastomoses in a rat model. Three days after surgery, the anastomoses with IGF-I coated sutures showed better anastomotic healing (histology appearance) and greater capacity to withstand biomechanical stress (busting pressure) than the control groups [23]. As an FDA-approved biologic, BMP-2 appears promising in promoting chondrogenesis and cartilage repair at pre-clinical setting [27,28,29]. The current study with BMP-2 coating on the resorbable sutures provides evidence to further support the theory that growth factors can be successfully introduced to a local tissue environment by coating suture material. Indeed, sutures are always needed to repair tissue tears/ruptures at clinical scenario. Coating the biologics to resorbable suture would be a feasible and useful strategy for local sustained delivery.

Although not on a large scale, this study has demonstrated that BMP-2 introduction to an avascular cartilage structure significantly promoted healing—with creation of cartilaginous tissue and not just a fibrous intermediary. The introduction of growth factors into an avascular cartilage model is not a novel idea and has been tried by Petersen et al. [24]. This group created meniscal tears in the avascular zone of sheep knees that were then repaired with suture coated in VEGF. They hypothesized that the avascular meniscus could be promoted to heal by the new growth of vasculature to the region stimulated by VEGF. Unfortunately, there was no difference observed between the control and test group animals despite histologic evidence that cells in the region had been stimulated by the VEGF. The current study clearly suggested that local release of BMP-2 improved cartilage healing, warrants further investigations to understand the molecular regulations during the meniscus repairs.

This study has several limitations. Most notably, the sample size appears small. Furthermore, the histologic specimens obtained could have had more thorough analysis including multiple stains and Polymerase chain reaction (PCR) analysis to look at gene expression. However, the application of the results in this study to the future of meniscal repair is nonetheless encouraging. Having demonstrated that BMP-2 coated suture can increase the rate of healing of an avascular cartilage structure in rats opens the door to more research. To start with, it would be helpful to examine the effect of BMP-2 on avascular cartilage on a larger scale and conduct more in-depth histologic analysis. For example, staining for gene expression within chondrocytes could be undertaken. Additionally, other growth factors including TGF-β, IGF-1 and Platelet-derived growth factor (PDGF) could be studied in a similar fashion [30,31]. In any event, the potential for improving the healing of avascular meniscal tissue is a worthwhile goal to work towards.

## 5. Conclusions

Although small in scope, this study has made several important points. First, the rat xyphoid process can serve as a model to study avascular cartilage. Secondly, this study corroborates previous evidence showing that growth factors can be delivered to local tissues effectively as a coating on suture material. Finally, this study demonstrates that BMP-2 coated suture has a positive effect on the healing of an avascular cartilage structure when compared to traditional suture.
